# Growth rate controlled synthesis of hierarchical Bi_2_S_3_/In_2_S_3_ core/shell microspheres with enhanced photocatalytic activity

**DOI:** 10.1038/srep04027

**Published:** 2014-02-07

**Authors:** Juan Zhou, Guohui Tian, Yajie Chen, Yunhan Shi, Chungui Tian, Kai Pan, Honggang Fu

**Affiliations:** 1 Key Laboratory of Functional Inorganic Material Chemistry Ministry of Education of the People's Republic of China Heilongjiang University, Harbin 150080 (P. R. China); 2 High-efficiency Conversion, College of Heilongjiang Province, School of Chemistry and Materials Science, Heilongjiang University, Harbin 150080, (P. R. China)

## Abstract

Core/shell heterostructure composite has great potential applications in photocatalytic field because the introduction of core can remarkably improve charge transport and enhance the electron-hole separation. Herein, hierarchical Bi_2_S_3_/In_2_S_3_ core/shell structured microspheres were prepared via a simple one-pot hydrothermal process based on different growth rate of the two kinds of sulphides. The results showed that, the as-prepared hierarchical Bi_2_S_3_/In_2_S_3_ core/shell heterostructure exhibits significant visible light photocatalytic activity for degradation of 2, 4-dichlorophenol. The introduction of Bi_2_S_3_ core can not only improve charge transport and enhance the electron-hole separation, but also broaden the visible light response. The hierarchical porous folwer-like shell of In_2_S_3_ could increase the specific surface area and remarkably enhanced the chemical stability of Bi_2_S_3_ against oxidation.

Recently, the design and synthesis of core/shell materials from nanoscale to microscale size have attracted much attention due to their unique structure-induced properties^1^,^2^,^3^. The interactions between the core and shell can significantly improve the overall performance of the core/shell system and even produce beneficial synergistic effects, which may bring a series of opportunities for their potential applications such as photoelectric devices, sensors and chemical catalysis^4^,^5^,^6^. In particular, hierarchical core/shell heterostructure photocatalysts have displayed superior photocatalytic efficiency because proper junctions formed between core and shell can efficiently accelerate charge separation and the large surface area can increase the active sites and light utilization^7^,^8^,^9^, so develop a simple method to prepare hierarchical core/shell heterostructures deserve researching.

Metal sulfides have been extensively investigated and proven to be a group of highly efficient catalysts for photochemical reactions. Typically, In_2_S_3_ nanostructure, a III-VI group sulfide, is known to crystallize in three polymorphic forms: α-In_2_S_3_, β-In_2_S_3_, and γ- In_2_S_3_. Of these, β- In_2_S_3_ is a n-type semiconductor with a band gap of 2.0–2.3 eV and is a potential candidate for photocatalytic applications because of its proper band gap for solar energy conversion^10^,^11^. Bi_2_S_3_ with a narrow bandgap (~1.3 eV) can be a good candidate semiconductor^12^,^13^. It has been used as a sensitizer due to its ability to absorb a large part of visible light up to 800 nm^14^,^15^. However, both of them exhibit relatively low photocatalytic activity. To improve the photocatalytic activity, it is highly desirable to fabricate In_2_S_3_/Bi_2_S_3_ core/shell heterostructure composite, which can efficiently promote charge separation and lead to enhanced activity. So the combination of In_2_S_3_ and Bi_2_S_3_ in a single core/shell composite could hold possible applications in energy conversion for the photodegradation reaction, which has not been reported yet.

In all cases to date, general synthesis strategies used to fabricate core/shell structures involve two main steps. The core powders are first prepared, and then coated with other materials (shell) to form a core–shell structure. It is difficult to achieve uniform coating with well-defined morphologies due to compatibility issues between the core and desired shell materials. Therefore, how to construct hierarchical heterogeneous core/shell structures via a facile one-spot route is still a significant challenge to material scientists.

Herein, we reported a facile route for growth rate controlled synthesis of hierarchical Bi_2_S_3_/In_2_S_3_ core/shell microspheres via a hydrothermal process. In this process, consecutive reactions of the sole sulfur source of L-cysteine with Bi and In salts lead to the formation of Bi_2_S_3_/In_2_S_3_ core/shell structures due to their significantly different reaction rate. The as-prepared core/shell hybrid has an interesting structure with uniform size consisting of a Bi_2_S_3_ sphere core and a nanosheet-based In_2_S_3_ shell. The special hierarchical structure, high light-harvesting capacity and the microscale core/shell heterostructure make it to be an excellent candidate for the degradation of 2, 4-dichlorophenol with enhanced photostability and photocatalytic efficiency.

## Results

**Figure 1:**
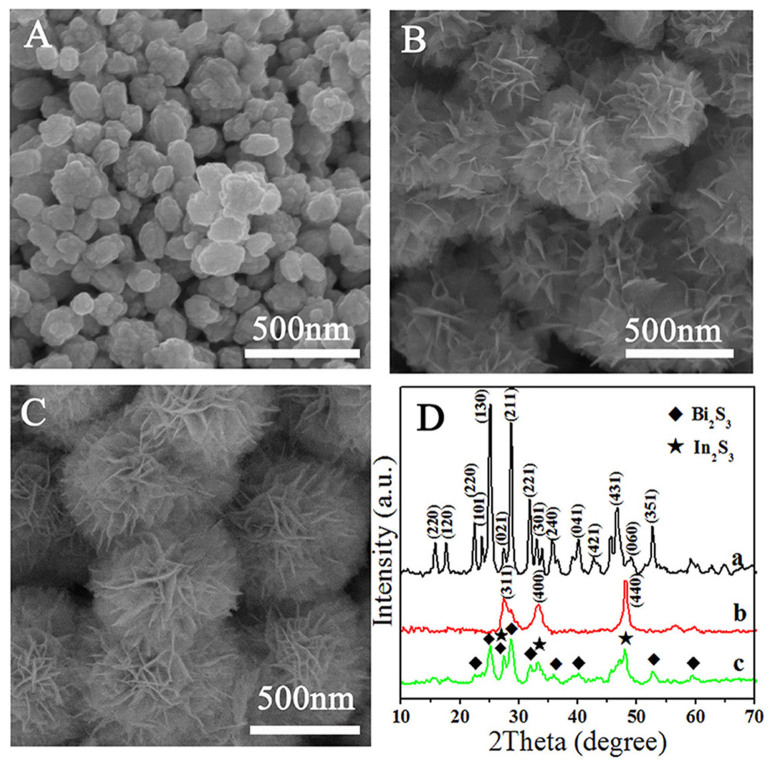
SEM images of Bi_2_S_3_ (A), In_2_S_3_ (B), hierarchical In_2_S_3_/Bi_2_S_3_ core/shell hybrid (In-Bi-30) (C), and XRD pattern (D) of the three samples: Bi_2_S_3_ (a), In_2_S_3_ (b), hierarchical Bi_2_S_3_/In_2_S_3_ core/shell hybrid (In-Bi-30) (c).

The morphology and the crystal phase information of the final products were investigated by SEM and XRD analysis, as shown in Figure 1. From the comparison of the SEM images we could clearly see that bare Bi_2_S_3_ is composed of nanospheres with an average diameter of 100–200 nm (Figure 1A). As for pure In_2_S_3_, flower-like structure with an average size of 400 nm could be easily obtained, and its surface is made up by thin nanosheets (Figure 1B). Interestingly, Figure 1C clearly indicates that Bi_2_S_3_/In_2_S_3_ core/shell composite is a uniform flower-like spherical superstructure composed of numerous intercrossed ultrathin nanosheets. The average diameter of these superstructures is about 500 nm, which is a little bit larger than the size of pure In_2_S_3_. This change infers that In_2_S_3_ nanosheets may grow along the core of Bi_2_S_3_ nanoparticles in the Bi_2_S_3_/In_2_S_3_ composites. The phase and purity of these samples were determined by XRD measurements (Figure 1D). The X-ray diffraction spectra of a and b shown in Figure 1D reveal the presence of Bi_2_S_3_ and In_2_S_3_, respectively. Correspondingly, all the peaks can be indexed to a pure orthorhombic phase Bi_2_S_3_ (JCPDS card No. 17-0320) and cubic β-In_2_S_3_ (JCPDS Card No. 32-0456). For hierarchical core/shell spherical superstructure (Line c in Figure 1D), diffraction peaks of In_2_S_3_ match with the reflections from the cubic β-In_2_S_3_ lattice planes of (311), (400) and (440), making it easy to reveal the presence of In_2_S_3_ phase. In contrast, other diffraction peaks can be attributed to orthorhombic phase Bi_2_S_3_. All the above results suggest that the intercrossed In_2_S_3_ nanosheet shell could *in situ* grow on the surface of Bi_2_S_3_ core.

**Figure 2:**
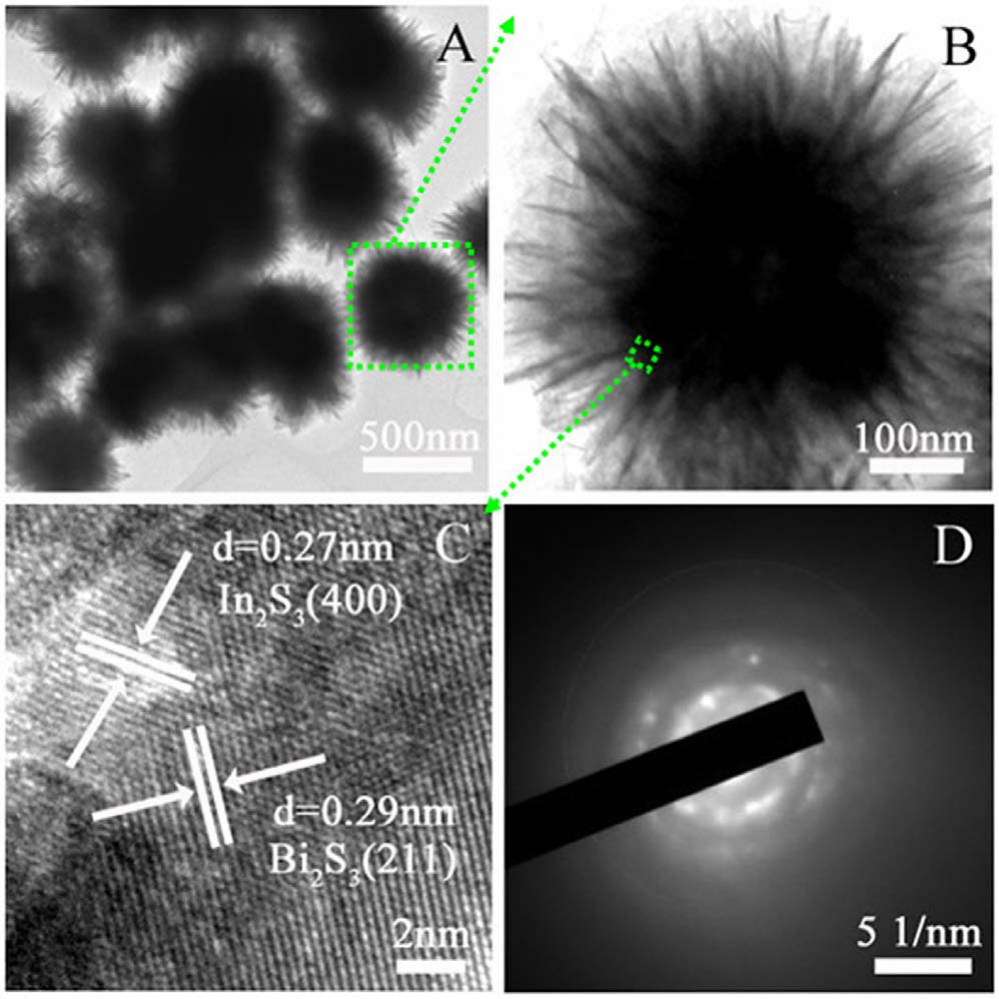
TEM (A, B), HRTEM (C) and the selected area electron diffraction pattern (D) images of the Bi_2_S_3_/In_2_S_3_ core/shell spheres (In-Bi-30).

Additionally, the more detailed structural information of the hierarchical Bi_2_S_3_/In_2_S_3_ composite was revealed using TEM and HRTEM. As shown in Figure 2A, ultrathin nanosheets grow uniformly on the surface of solid sphere core and the thickness of the nanosheets is estimated to be 3–5 nm measured from the edges (Figure 2B). The HRTEM image of the core-shell structure (Figure 2C) reveals that the interplanar spacing of 0.29 nm corresponds to the (211) plane of Bi_2_S_3_, while 0.27 nm corresponds to the (400) plane of cubic β-In_2_S_3_, which implying the formation of Bi_2_S_3_/In_2_S_3_ heterostructure. The selected area electron diffraction pattern (SAED) image in Figure 2D further proves its polycrystalline mixed-phase nature. It should be noted that our as-synthesized Bi_2_S_3_/In_2_S_3_ core/shell spheres have a small diameter and the constituent nanosheets are very thin, which would contribute to the light-harvesting in the photocatalytic reaction.

**Figure 3:**
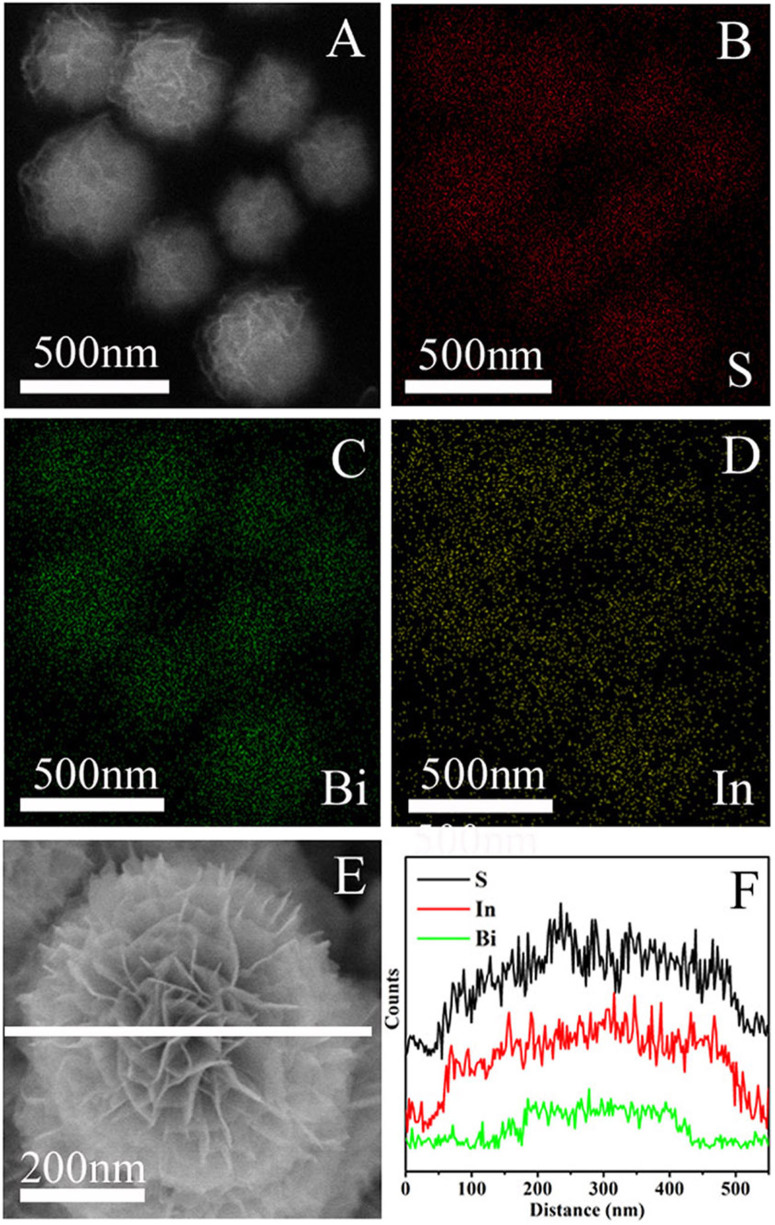
SEM image (A) of the hierarchical Bi_2_S_3_/In_2_S_3_ core/shell spheres (In-Bi-30) and the corresponding EDS mapping of S (B, red), Bi (C, green) and In (D, yellow); single Bi_2_S_3_/In_2_S_3_ core/shell sphere SEM image (E) and corresponding EDS line scan profiles (F) of S (K edge), In (L edge) and Bi (L edge) across the sphere displayed in the (E).

To further accurately investigate the elemental composition as well as the spatial uniformity of the elemental distribution, X-ray energy dispersive spectrometry (EDS) was carried out on Bi_2_S_3_/In_2_S_3_ spheres (Figure 3). The EDS mapping images indicate the coexistence of S, Bi and In elements in the submicrosphere (Figure 3B–D). The S element mapping (Figure 3B) confirms the homogeneous distribution among the whole architecture, but for the other two elements, the diameter of Bi element mapping distribution is smaller (Figure 3C) than that of In element mapping distribution (Figure 3D), which confirms that the In_2_S_3_ nanosheets are densely and uniformly decorated on the surface of the Bi_2_S_3_ cores. Besides, energy dispersive X-ray spectroscopy (EDS) line scans on single submicrosphere (Figure 3F) show that the synthesized flower-like Bi_2_S_3_/In_2_S_3_ spheres are indeed unique structures with the Bi_2_S_3_ cores buried inside the In_2_S_3_ shells.

**Figure 4:**
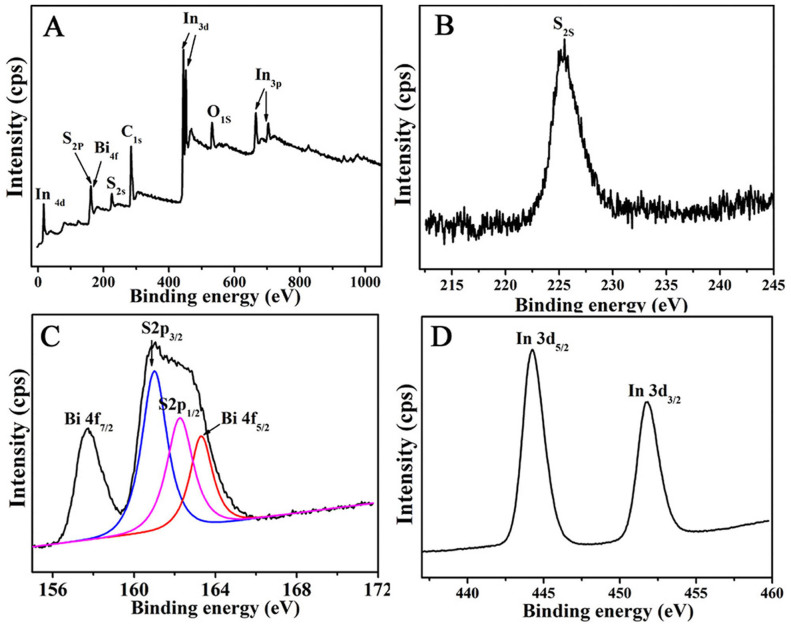
The survey XPS spectrum (A) of the hierarchical Bi_2_S_3_/In_2_S_3_ core/shell composite (In-Bi-30) and the high-resolution scan of S2s (B), Bi4f and S2p (C) and In 3d peaks (D) of the hierarchical Bi_2_S_3_/In_2_S_3_ core/shell composite (In-Bi-30).

The surface valence state and the chemical composition of the hierarchical Bi_2_S_3_/In_2_S_3_ core/shell composite were further characterized by XPS. Figure 4A clearly indicates that the product is mainly composed of S, Bi and In elements (C and O signals come from the reference sample and absorbed oxygen). High-resolution scans of the three elements reveal several prominent peaks centered at around 225.2 (Figure 4B), 160.5, 157.75 (Figure 4C), and 444.8 eV (Figure 4D), which can be accordingly assigned to binding energies of S2s, S2p_3/2_, Bi4f_7/2_, and In3d_5/2_^16^,^17^. Moreover, phenomena of spin orbit separation between Bi 4f_7/2_ and Bi 4f_5/2_ peaks (5.30 eV), S2p_3/2_ and S2p_1/2_ peaks (1.25 eV) (Figure 4C), and In 3d_5/2_ and In 3d_3/2_ peaks (7.5 eV) (Figure 4D) suggest the existence of S^2+^, Bi^3+^ and In^3+^ in the final product, which is in agreement with previous reports^18^,^19^.

For highly efficient photocatalyst, light absorption range and intensity are important. The optical absorption properties of Bi_2_S_3_, In_2_S_3_ and the hierarchical Bi_2_S_3_/In_2_S_3_ core/shell composite are displayed in the Figure S1. According to the UV–vis diffuse reflectance spectra, both Bi_2_S_3_ and In_2_S_3_ present the photoresponse property from UV light region to visible light region. Compared with bare Bi_2_S_3_, the light absorption ability of the core/shell material is enhanced after In_2_S_3_ was introduced, which has strong absorption in nearly the whole range of visible light. This can be attributed to the small band gap and synergistic affect of the two compositions. Taking into account the efficient use of visible light in a large part of the solar spectrum, we believe that this photocatalyst, with its long wavelength absorption band, is an attractive photocatalyst for pollutant degradation.

**Figure 5:**
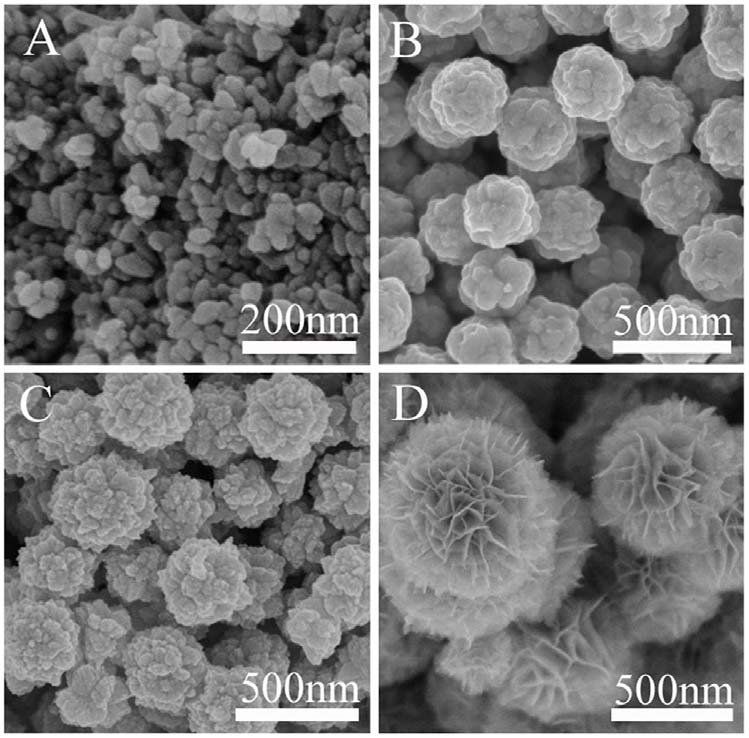
SEM images of the products obtained at 20 min (A), 1 h (B), 3 h (C) and 9 h (D) in one-pot reaction.

To investigate the formation process of the hierarchical Bi_2_S_3_/In_2_S_3_ core/shell spheres, samples prepared at different reaction times were collected and investigated by SEM. As shown in Figure 5A, at the early reaction stage (20 min), the product was composed of nanoparticles (15–25 nm). The corresponding EDS spectrum (Figure S2A a) inferred that these nanoparticles were composed of Bi and S elements, indicating the fast nucleation of Bi_2_S_3_ under hydrothermal condition. By prolonging the reaction time to 1 h, small nanoparticles congregated into compact submicrospheres (200–300 nm, Figure 5B) rapidly, and there were numerous small protuberances on the surface of the submicrospheres, which would provide many high energy sites for further growth^20^. The EDS analysis (Figure S2A b) inferred the submicrosphere was also the phase of Bi_2_S_3_. As the reaction proceeded (Figure 5C), In^3+^ and S^2−^ could get enough energy to crystallize into In_2_S_3_ nanoparticles on the surface of Bi_2_S_3_ core. Meanwhile, the presence of In element (Figure S2A c) also proved the appearance of In_2_S_3_ in the composite. As the mass diffusion and Ostwald ripening process progressed, In_2_S_3_ nanosheets shell formed (Figure 5D), accompanied by their self-organization onto the Bi_2_S_3_ core to form hierarchical structure through a nucleation-aggregation-deposition pathway after the reaction was carried out for 9 h (Figure S2A d), and the average size of these spheres was about 500 nm. Furthermore, for the samples prepared from different reaction time, the content of In element increased along with prolonging the reaction time according to the EDS results (Figure S2A and Table S1).

Corresponding X-ray diffraction (XRD) patterns (Figure S2B) show the presence of crystalline Bi_2_S_3_ as the reaction time ranging from 20 min to 9 h. The diffraction peaks of In_2_S_3_ can hardly be found when reaction time was short (Figure S2B a and b), but the diffraction peaks of In_2_S_3_ started to emerge as the experiment was conducted for 3 h (Figure S2B c), and the diffraction peak intensity increased with the extension of reaction time (Figure S2B d). Furthermore, the peak intensity of Bi_2_S_3_ decreased to some extent as time prolonged, which further inferred that Bi_2_S_3_ was wrapped by the In_2_S_3_ shell and matched with the SEM (Figure 5) and EDS (Figure S2A) observations.

**Figure 6:**
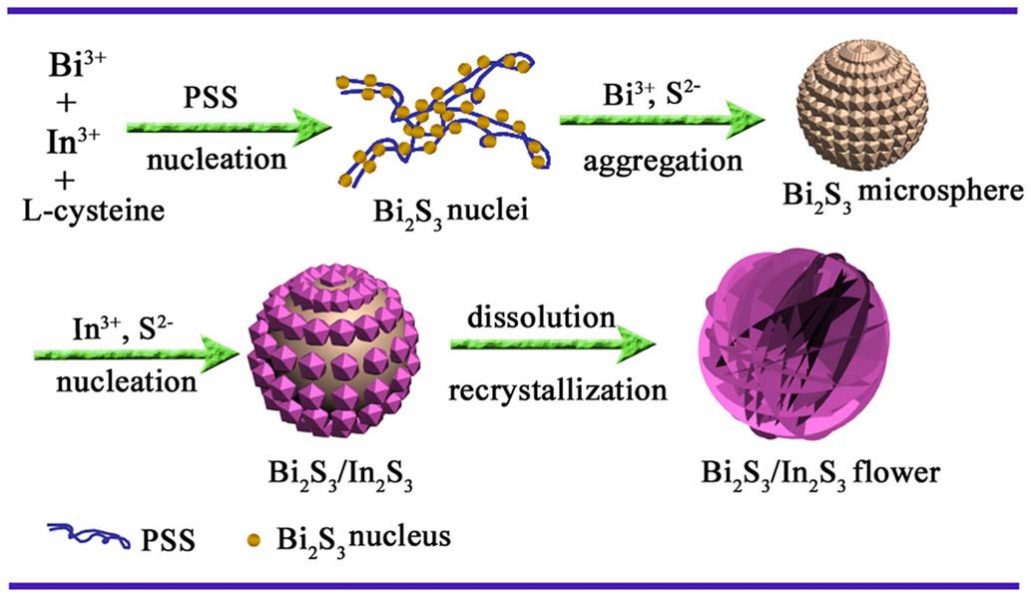
Schematic illustration of the morphological evolution process of the hierarchical Bi_2_S_3_/In_2_S_3_ core/shell spherical structure.

Based on the above experimental results and analysis, the probable morphology evolution process of the hierarchical Bi_2_S_3_/In_2_S_3_ core/shell spheres is illustrated in Figure 6. Compared with the general S sources (such as S powder, thiourea, Na_2_S and thioacetamide) used in the synthesis of semiconductor metal sulfides^21^, L-cysteine is an ordinary acidic amino acid biomolecule, which may avoid the cations hydrolyzing intensively, and the thiol groups can slowly supply S^2−^ in solution during the hydrothermal process. In the L-cysteine molecule, there are many functional groups, such as -NH_2_, -COOH, and -SH, which have a strong tendency to coordinate with inorganic cations and metals^22^. Besides, it has been reported that poly (sodium-p-styrenesul-fonate) (PSS) is an anionic surfactant with a long chain structure, which can provide many coordination sites for cations and other groups^23^, this has also been proved by our previous report^24^. In our experiment, the presence of an appropriate amount of surfactant PSS was crucial for the formation of this unique hierarchical Bi_2_S_3_/In_2_S_3_ core/shell spheres structure with small size. Our contrast experiment (Figure S3) result showed that without PSS, the size of the product was not uniformly and much larger than Bi_2_S_3_/In_2_S_3_ core/shell spheres (In-Bi-30). Notably, there were different morphologies in the final product, which indicated that In_2_S_3_ and Bi_2_S_3_ can't grow intimately to form core/shell structure. Therefore, under the help of mutual coordination between PSS and L-cysteine, Bi^3+^ and In^3+^ can coordinate with L-cysteine to form initial precursor complexes on the long chain structure of PSS^25^,^26^. At elevated reaction temperature, L-cysteine hydrolyzed to release H_2_S with the assistance of water. Because the *K*sp of Bi_2_S_3_ (1.0 × 10^−97^) is much smaller than that of In_2_S_3_ (5.7 × 10^−73^)^27^, it is expected that Bi_2_S_3_ will preferentially deposit and form crystal seeds along the PSS chain before In_2_S_3_ (Figure 5A). Since PSS long chains were flexible enough to interweave, the Bi_2_S_3_ nuclei tended to aggregate into larger spheres along with the continuous crystallization of Bi_2_S_3_ (Figure 5B). As the reaction proceeded, nucleation process of In_2_S_3_ was initialized when Bi^3+^ was depleted (Figure 5C). The as-produced In_2_S_3_ nanoparticles preferentially deposited on the surfaces of the preformed Bi_2_S_3_ spheres via epitaxial growth process to reduce their surface energy^18^. Further extending the reaction time, the outward In_2_S_3_ nanoparticles could get enough energy to dissolve into the solution and spontaneously nucleated onto these protuberances. As the mass diffusion and Ostwald ripening process progressed, the nanosheets formed accompanied with their self-organization into the flower-like structure^28^. Finally, the submicrospheres were constructed into hierarchical Bi_2_S_3_/In_2_S_3_ core/shell spherical structures (Figure 5 D).

## Discussion

Compared to the conventional synthetic methods, this one-spot synthetic route is simple and easy to achieve uniform coating with hierarchical nanosheets due to compatibility issues between the Bi_2_S_3_ core and In_2_S_3_ shell. Moreover, the In_2_S_3_ shell thickness and morphology can be easily controlled by altering the ratio of In_2_S_3_ in the Bi_2_S_3_/In_2_S_3_ architecture and reaction time. In order to obtain a higher efficient photocatalytic catalyst, we adjusted the ratio of In_2_S_3_ in the Bi_2_S_3_/In_2_S_3_ core/shell architectures, the morphologies were shown in Figure S4. When the content of In_2_S_3_ was low, the nanosheets on the surface of the microspheres were not obviously and the size was smaller (Figure S4A) than the In-Bi-30 sample (Figure S4B), indicating the formation of nanosheets needed sufficient amount of In_2_S_3_. Obviously, without fluffy nanosheets on the surface, the multiple light reflection would be reduced in the composite, which will reduce light-harvesting and thus decrease the quantity of photogenerated electrons and holes^29^. However, excess In_2_S_3_ (In-Bi-50) could make the nanosheets much thicker and radius much larger (Figure S4C), which would lead to the decrease of BET surface area, just as shown in Figure S5, Table S2. The corresponding BET surface areas and porous structures of different samples were investigated using nitrogen adsorption–desorption experiments (Figure S5). The pore-size distributions (the inset in Figure S5) of the samples indicate a pore-size distribution from 2 to 4 nm, confirming the presence of a large number of mesopores. Compared with Bi_2_S_3_ nanospheres, the BET surface areas of core/shell Bi_2_S_3_/In_2_S_3_ samples were improved (Table S2), especially the value of In-Bi-30, which was close to the BET surface area of In_2_S_3_ (76 m^2^/g). This can be attributed to its smaller particle size, ultrathin nanosheets and the presence of large quantity of mesoporous. The large surface area for heterogeneous photocatalysis can provide more surface active sites for the adsorption of reactant molecules, which will make the photocatalytic process more efficient^30^. But too thick a shell of the composite (In-Bi-50) caused the size of microspheres to become larger and the BET surface area smaller, meanwhile extended the diffusion length of charge-carrier transport in the Bi_2_S_3_/In_2_S_3_ core/shell hybrid to increase bulk combination in In_2_S_3_ or Bi_2_S_3_, which are not beneficial for the photocatalytic reaction. So we predicted that the hierarchical flower-like core/shell Bi_2_S_3_/In_2_S_3_ (In-Bi-30) with heterostructure and ultrathin nanosheets will have higher photocatalytic ability than other samples.

**Figure 7:**
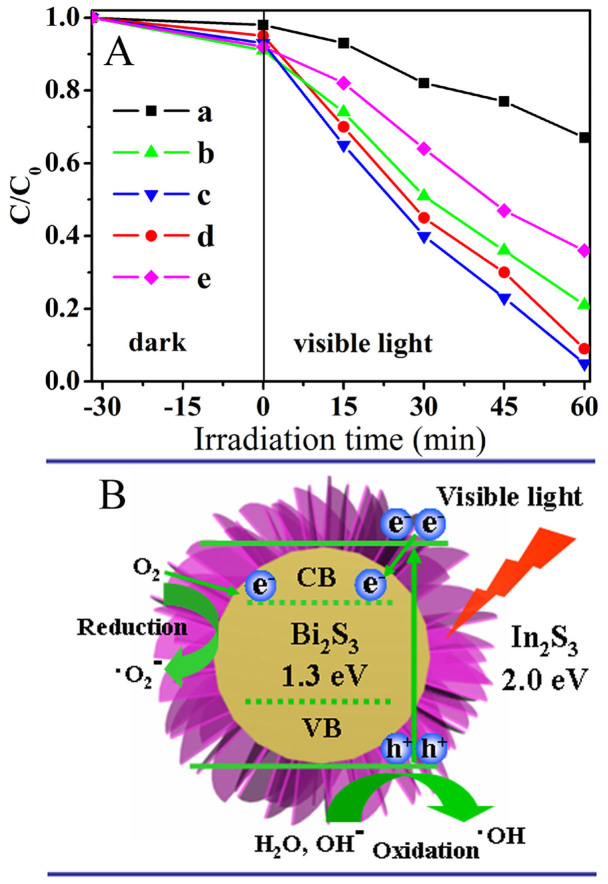
(A) Photocatalytic degradation of 2, 4-dichlorophenol by different photocatalysts under visible light irradiation: Bi_2_S_3_ (a), In-Bi-10 (b), In-Bi-30 (c), In-Bi-50 (d) In_2_S_3_ (e). *C* and *C*_0_ are the initial concentration after the equilibrium adsorption and the reaction concentration of 2, 4-dichlorophenol, respectively. (B) Schematic illustration showing the reaction mechanism for photocatalytic degradation of organic pollutants over the Bi_2_S_3_/In_2_S_3_ core/shell composite.

Considering the core/shell architectures with special hierarchical nanocomposites are advantageous to photocatalytic application, we investigate the photocatalytic properties of as-prepared hierarchical Bi_2_S_3_/In_2_S_3_ core/shell nanostructures by the degradation of 2, 4-dichlorophenol under visible light irradiation in an aqueous solution, together with that of Bi_2_S_3_ and In_2_S_3_ for the purpose of comparison. Before the photocatalytic reaction, the dark adsorption experiments were performed. As shown in Figure 7A, the dark adsorption amounts of pure In_2_S_3_ and In-Bi-30 were high, which associate with their high BET surface areas. After irradiation, the plots for the concentration changes of 2, 4-dichlorophenol determined from its characteristic absorption peak over different catalysts are altered. It can be seen that all the In_2_S_3_/Bi_2_S_3_ core/shell composites show higher photocatalytic activities than individual Bi_2_S_3_ and In_2_S_3_ under identical experimental conditions. As discussed above, the hierarchical core/shell structure of the In_2_S_3_/Bi_2_S_3_ composites improves the light absorption and facilitates the efficient separation of photogenerated carriers, which can be considered as main reasons for the enhancement of photocatalytic activities. The photocatalytic mechanism was shown in Figure 7B. Under visible-light illumination, photogenerated electrons are excited from the value band (VB) to the conduction band (CB) of In_2_S_3_. Because the CB of In_2_S_3_ is slightly higher than that of Bi_2_S_3_, the photoexcited electrons in the CB of In_2_S_3_ can be transferred to Bi_2_S_3_ in the core/shell hybrid nanostructure easily, creating positive holes in the VB of In_2_S_3_. Importantly, the In_2_S_3_ shell is made up of ultrathin nanosheets with intersection, there exist lots of porous channels, the oxygen could permeate through the shell to the core of Bi_2_S_3_. Since the E_CB_ potential of Bi_2_S_3_ (−0.76 eV vs NHE) is more negative than E_0_ (O_2_/·O_2_-) (−0.046 eV vs NHE), the electrons left on the E_CB_ of Bi_2_S_3_ could reduce O_2_ to ·O_2_- through one-electron reducing reaction^31^. Meanwhile, due to the high oxidizing potential, the formed holes may react with H_2_O or OH^−^ in the solution, generating hydroxyl (**·**OH) radicals adsorbed at the surface^32^,^33^. All the radicals can react with organic chemicals in the solution and improve the photoactivity. It should be pointed out that the amount of In_2_S_3_ has obvious influence on the photocatalytic ability in the present material system (Figure 7A b–d). When the amount of In_2_S_3_ is relatively low (In-Bi-10), the small surface area of Bi_2_S_3_/In_2_S_3_ core/shell nanostructure provides relatively low surface active sites for the adsorption of reactant molecules. Increase the amount of In_2_S_3_, hierarchical core–shell structure was formed (In-Bi-30), the degradation rates of 2, 4-dichlorophenol reached the maximum value (95%, Figure 7A c). Further adding the In_2_S_3_ content also reduced the catalytic efficiency of the Bi_2_S_3_/In_2_S_3_ core/shell nanostructures, suggesting that a too thick In_2_S_3_ shell was unfavorable to the degradation of 2, 4-dichlorophenol owing to the increased recombination of excited holes and electrons. These systematic changes further demonstrate the favorable role of the core/shell-structured Bi_2_S_3_/In_2_S_3_ catalyst in the preferential degradation of 2, 4-dichlorophenol by the photogenerated holes and electrons. In addition, the inter-meshed nanosheets of hierarchical superstructure (In-Bi-30) can allow multiple reflections of visible light, which enhances light-harvesting and thus increases the quantity of photogenerated electrons and holes available to participate in the photocatalytic degradation of 2, 4-dichlorophenol^34^.

To further prove the mechanism of the photocatalysis, the formation of OH on the surface of photocatalysts was detected by the fluorescence technique using terephthalic acid (TA) as a probe molecule. It is well known that _•_OH reacts with terephthalic acid (TA) in basic solution to generate 2-hydroxy-terephthalic acid (TAOH), which emits a unique fluorescence signal with the peak centered at 426 nm^35^. The fluorescence intensity of TAOH is proportional to the amount of**·**OH produced on the surface of photocatalysts. The maximum emission intensity in fluorescence spectra was recorded at 425 nm by the excitation at 315 nm, and the results are shown in Figure S6. It clearly demonstrates that the photoexcited holes are powerful enough to oxidize surface-adsorbed hydroxy groups and water molecules to generate _•_OH radicals. Obviously, the maximum number of _•_OH radicals is formed by using the Bi_2_S_3_/In_2_S_3_ core/shell photocatalyst (In-Bi-30) in the photoreaction process, this result is in good agreement with the result of photodegradation of 2, 4-dichlorophenol. Hence, the photocatalytic activity has a positive correlation with the formation rate of _•_OH radicals^36^. Besides, the trapping experiments of active species during the photocatalytic reaction were performed. Isopropanol (IPA) and benzoquinone (BQ) acted as the scavengers for **·**OH and ·O_2_-, respectively^37^. Figure S7 displays the results of different scavengers on the photodegradation rate over the sample of Bi_2_S_3_/In_2_S_3_ core/shell nanostructure (In-Bi-30) with 30 min irradiation. It can be seen that the addition of IPA and BQ in the reaction solution both have apparent effect on the photocatalytic activity, suggesting that _•_OH and ·O_2_- are the main oxygen active species involved in the 2, 4-dichlorophenol photocatalytic process under visible light irradiation. All of the above results could well support the proposed photocatalytic mechanism in Figure 7B.

Metal sulfides are usually not stable during the photocatalytic reaction and subjected to photocorrosion^38^,^39^. In our experiment, the stability of the Bi_2_S_3_/In_2_S_3_ core/shell structure (In-Bi-30) and pure Bi_2_S_3_ and In_2_S_3_ were evaluated by performing the cycling experiments under the same conditions (Figure S8). After four recycles, the photocatalytic degradation rate of sample Bi_2_S_3_ and In_2_S_3_ decreases gradually, however the Bi_2_S_3_/In_2_S_3_ core/shell structure (In-Bi-30) does not exhibit evident loss of activity, indicating the better stability of this Bi_2_S_3_/In_2_S_3_ core/shell composite. The high stability of the Bi_2_S_3_/In_2_S_3_ core/shell structure is attributed to the close interaction between Bi_2_S_3_ and In_2_S_3_ solid solution, which favors the transfer of the photogenerated electrons from the conduction band (CB) of In_2_S_3_ to Bi_2_S_3_ conduction band (CB), as shown in Figure 7B. This space separation of the photogenerated electrons and holes is beneficial for preventing the reduction of In^3+^ and Bi^3+^.

In summary, we demonstrated a facile one-pot hydrothermal method to synthesize hierarchical Bi_2_S_3_/In_2_S_3_ core/shell microspheres according to the different growth rate of the two kinds of sulphides. Moreover, their performance studies indicated the hierarchical Bi_2_S_3_/In_2_S_3_ core/shell microspheres exhibit higher photocatalytic activity than the pure Bi_2_S_3_ and In_2_S_3_ for the degradation of 2, 4-dichlorophenol due to the efficient synergistic effect resulted from the interaction between Bi_2_S_3_ core and hierarchical In_2_S_3_ shell. The synergistic activity was also investigated through the measurement of _•_OH and ·O_2_- radicals produced by Bi_2_S_3_/In_2_S_3_ core/shell microspheres. It is considered that this facile and promising synthetic strategy can be extended to prepare a wide variety of functional nanohybrids for potential applications in waste water treatment, solar cells and so on.

## Methods

### Preparation of Bi_2_S_3_/In_2_S_3_ core/shell microspheres

In a typical experiment, 0.3 g Bi(NO_3_)_3_·5H_2_O, 0.15 g poly (sodium-p-styrenesul-fonate) (PSS), a specified quality of In(NO_3_)_3_·4.5H_2_O and L-cysteine were added into 25 mL distilled water and stirred for 30 min at room temperature. Then, the obtained yellow solution was transferred to a 50 mL Teflon-lined stainless steel autoclave, which was heated to 150°C and maintained for 9 h. After cooling, the as-synthesized brown products were rinsed with distilled water, absolute ethanol, respectively, and dried at 60°C overnight. The molar ratios of In^3+^ to Bi^3+^ were 10%, 30%, and 50%, and the resulting samples were labeled as In-Bi-10, In-Bi-30 and In-Bi-50, respectively. For comparison, bare In_2_S_3_ and Bi_2_S_3_ were obtained under the same experimental conditions in the absence of Bi(NO_3_)_3_·5H_2_O and In(NO_3_)_3_·4.5H_2_O, respectively.

### Materials characterization

Structure and morphology of the product was investigated by Scanning electron microscopy (SEM, Hitachi S-4800, Japan), SEM-EDS analyses were carried out with a Hitachi S-4800 SEM equipped with an EDAX energy dispersive X-ray analyzer. Transmission electron microscopy (TEM, JEOL 2100, Japan), and powder X-ray diffraction (XRD, Bruker D8 Advance using CuKa radiation). The UV-visible diffuse reflectance spectra of films were obtained using a UV-visible spectrophotometer (Shimadzu UV-2550). The electronic states of elements in the sample were analyzed using X-ray photoelectron spectroscopy (XPS, Kratos-AXIS UL TRA DLD, Al Ka X- ray source). The specific surface areas of the materials were calculated using the Brunauer–Emmett–Teller (BET) method.

### Photocatalytic properties study

The photodegradation experiments were performed in a slurry reactor containing 100 mL of 50 mg/L 2, 4-dichlorophenol and 0.05 g of catalyst. An 150 W xenon lamp (Institute of Electric Light Source, Beijing) was used as the solar-simulated light source, and visible light was achieved by utilizing a UV cut filter (*λ* ≥ 420 nm). Prior to light irradiation, the suspension was kept in the dark under stirring for 30 min to ensure the establishing of an adsorption/desorption equilibrium. Oxygen flow was employed in all experiments as oxidant. Adequate aliquots (5 mL) of the sample were withdrawn after periodic interval of irradiation, and centrifuged at 10000 rpm for 5 min, then filtered through a Milipore filter (pore size 0.22 μm) to remove the residual catalyst particulates for analysis. The filtrates were analyzed using a UV-vis spectrophotometer (Shimadzu UV-2550).

In order to detect the active species during the photocatalytic reaction, hydroxyl (_•_OH) radicals produced by the photocatalysts under visible light irradiation were measured by the fluorescence method on a Fluoromax-4 Spectrophotometer (Horiba Jobin Yvon) using terephthalic acid (TA) as a probe molecule. The _•_OH radical trapping experiments were carried out using the following procedure: A 5 mg portion of the sample was dispersed in 30 mL of a 5 × 10^−4^ M TA aqueous solution in a diluted NaOH aqueous solution (2 × 10^−3^ M). The resulting suspension was then exposed to visible light irradiation for 15 min. 2 mL of the suspension was collected and centrifuged to measure the maximum fluorescence emission intensity with an excitation wavelength of 315 nm. Besides, isopropanol (IPA, 10 mM), and benzoquinone (BQ, 6 mM) were added into the 2, 4-dichlorophenol solution to capture hydroxyl radicals (**·**OH) and the superoxide radicals (·O_2_-), respectively, followed by the photocatalytic activity test.

## Author Contributions

J.Z. performed synthesis experiments, G.H.T. and H.G.F. designed the experiment. Y.J.C. and Y.H.S. contributed in material characterization and discussion. C.G.T. and K.P. carried out photocatalytic evaluation and discussion. J.Z. and G.H.T. wrote the manuscript.

## Supplementary Material

Supplementary Information dataset 1
