# Supplementary material for: Growth rate controlled synthesis of hierarchical Bi2S3/In2S3 core/shell microspheres with enhanced photocatalytic activity
Source: Sci Rep. 2014 Feb 7;4:4027. doi: 10.1038/srep04027 (PMC3916892; doi:10.1038/srep04027)
Supplement: Supplementary Information — dataset 1 [file srep04027-s1.doc]

Subject areas

NANOSCALE MATERIALS, ENVIRONMENTAL CHEMISTRY

Correspondence and requests for materials should be addressed to H. G. Fu (fuhg@ vip.sina.com) or G. H. Tian (tiangh@hlju.edu.cn)

Growth rate controlled synthesis of hierarchical Bi_2_S_3_/In_2_S_3_ core/shell microspheres with enhanced photocatalytic activity

Juan Zhou,^1^ Guohui Tian,^1,2^ Yajie Chen,^1^ Yunhan Shi,^1^ Zhiyu Ren,^1^ Kai Pan^1^ and Honggang Fu^1^

^1^Key Laboratory of Functional Inorganic Material Chemistry Ministry of Education of the People’s Republic of China Heilongjiang University, Harbin 150080 (P. R. China) Fax:(+86)451-8666-1259, ^2^High-efficiency Conversion, College of Heilongjiang Province, School of Chemistry and Materials Science, Heilongjiang University, Harbin 150080, (P. R. China).

**Table S1** Samples and the corresponding content of every element

| Sample | Element | Weight (%) | Atom (%) |
| --- | --- | --- | --- |
| A | Bi | 81.35 | 40.04 |
|  | In | 0 | 0 |
|  | S | 18.65 | 59.96 |
| B | Bi | 80.43 | 38.63 |
|  | In | 0 | 0 |
|  | S | 19.57 | 61.37 |
| C | Bi | 76.16 | 37.75 |
|  | In | 6.39 | 5.76 |
|  | S | 17.45 | 56.49 |
| D | Bi | 44.03 | 17.36 |
|  | In | 33.07 | 23.69 |
|  | S | 22.90 | 58.95 |

**Table S2** Some physical characteristics of different samples

| Sample | Bi_2_S_3_ | In-Bi-10 | In-Bi-30 | In-Bi-50 | In_2_S_3_ |
| --- | --- | --- | --- | --- | --- |
| BET surface area (m^2^/g) | 41 | 51 | 72 | 63 | 76 |
| Pore diameter (nm) | 2.3 | 2.7 | 2.4 | 2.5 | 2.3 |
| Adsorption rate of chlorophenol (C/C_0_) | 0.02 | 0.05 | 0.09 | 0.07 | 0.08 |


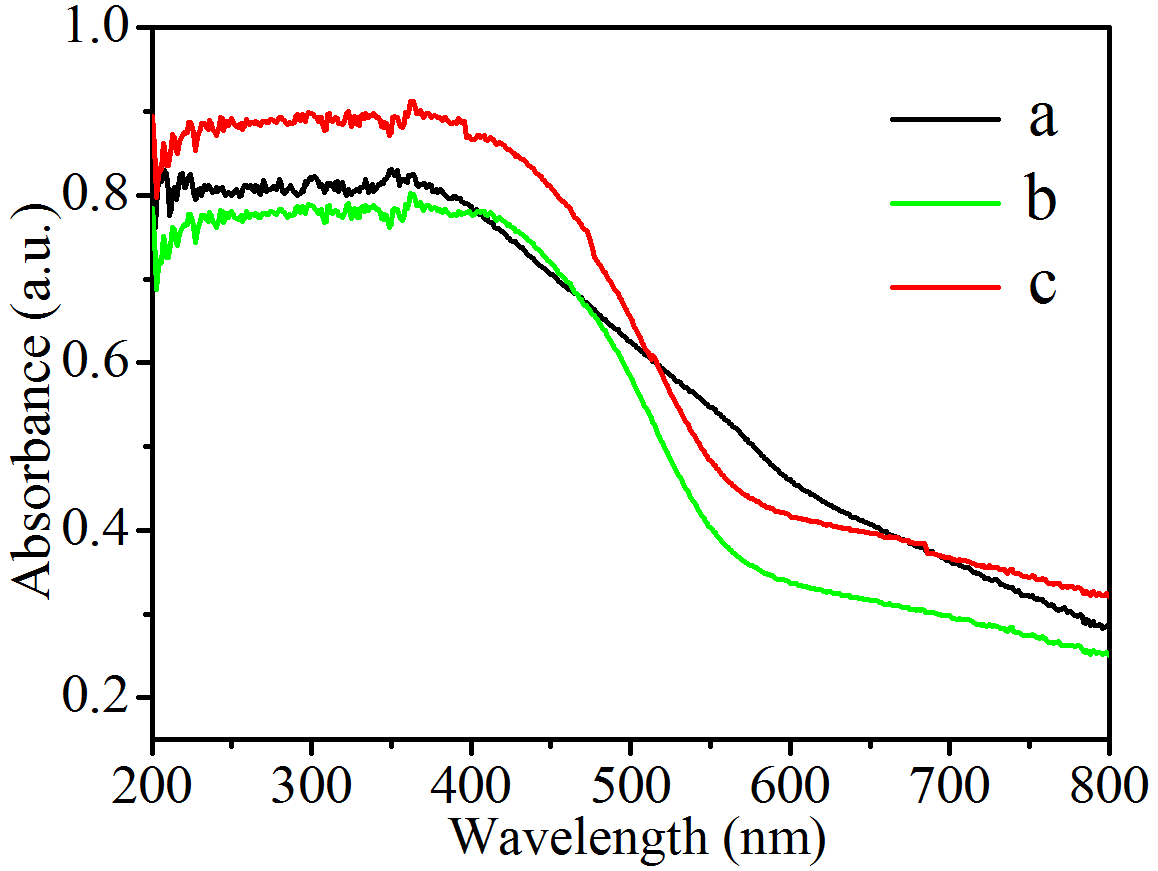


**Figure S1.** UV−vis diffuse reflectance spectra of the as-prepared samples: Bi_2_S_3_ (a), In_2_S_3_ (b), hierarchical Bi_2_S_3_/In_2_S_3_ core/shell composite (In-Bi-30) (c).


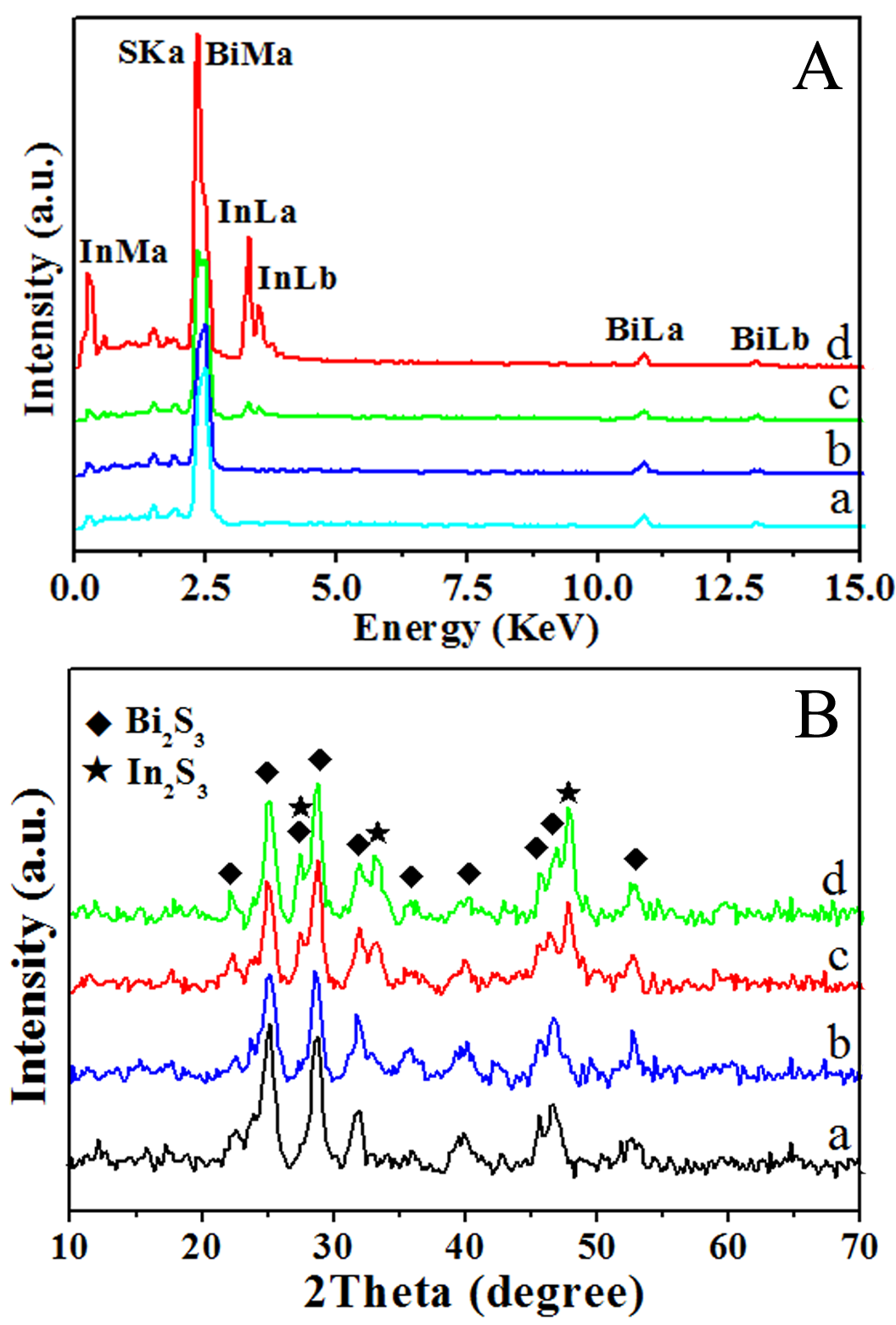


**Figure S2.** EDS spectra (A) and XRD patterns (B) of the samples obtained at different reaction time: 20 min (a), 1 h (b), 3 h (c) and 9 h (d).


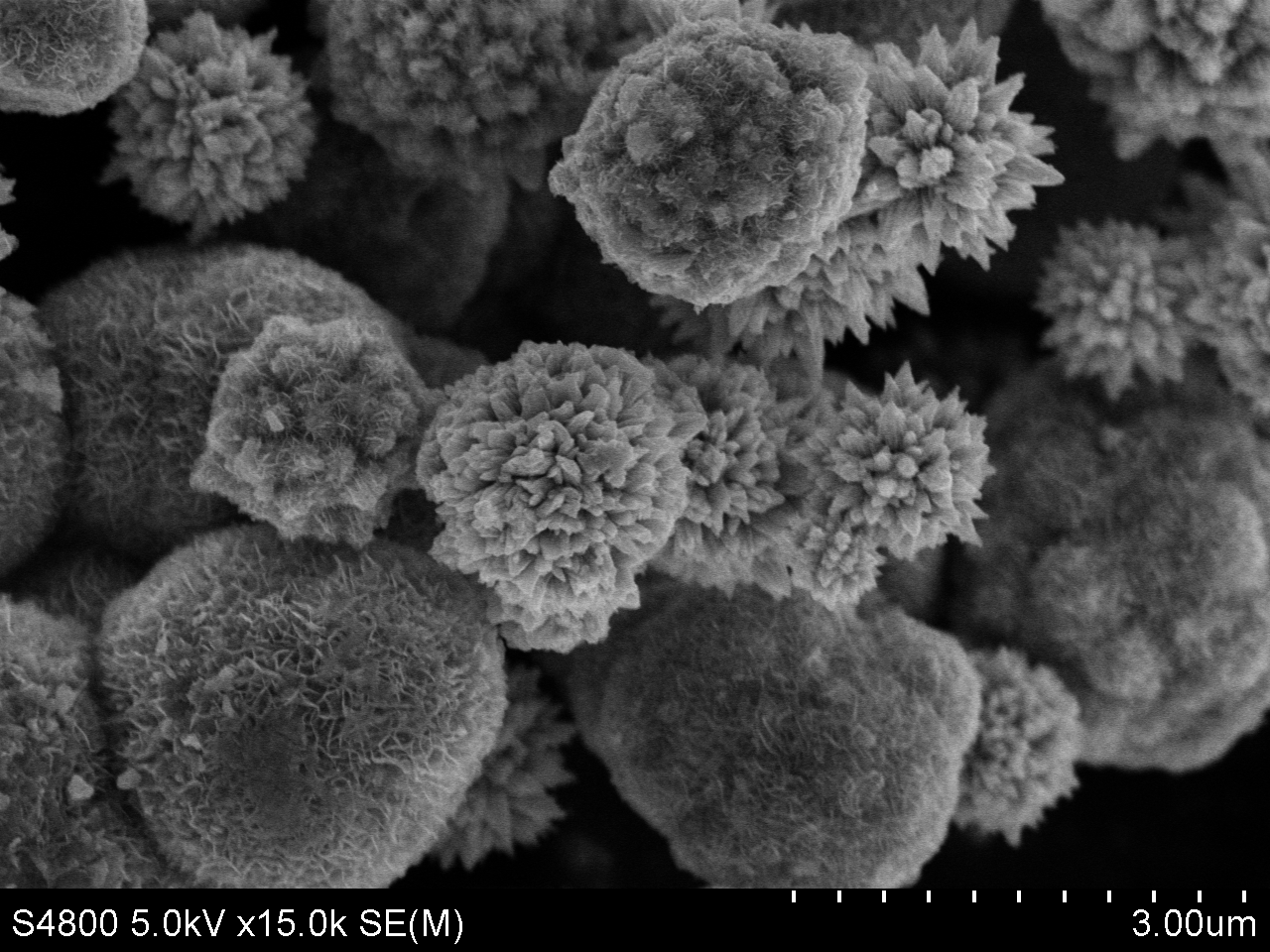


**Figure S3.** SEM image of the product prepared from hydrothermal reaction in the absence of PSS.


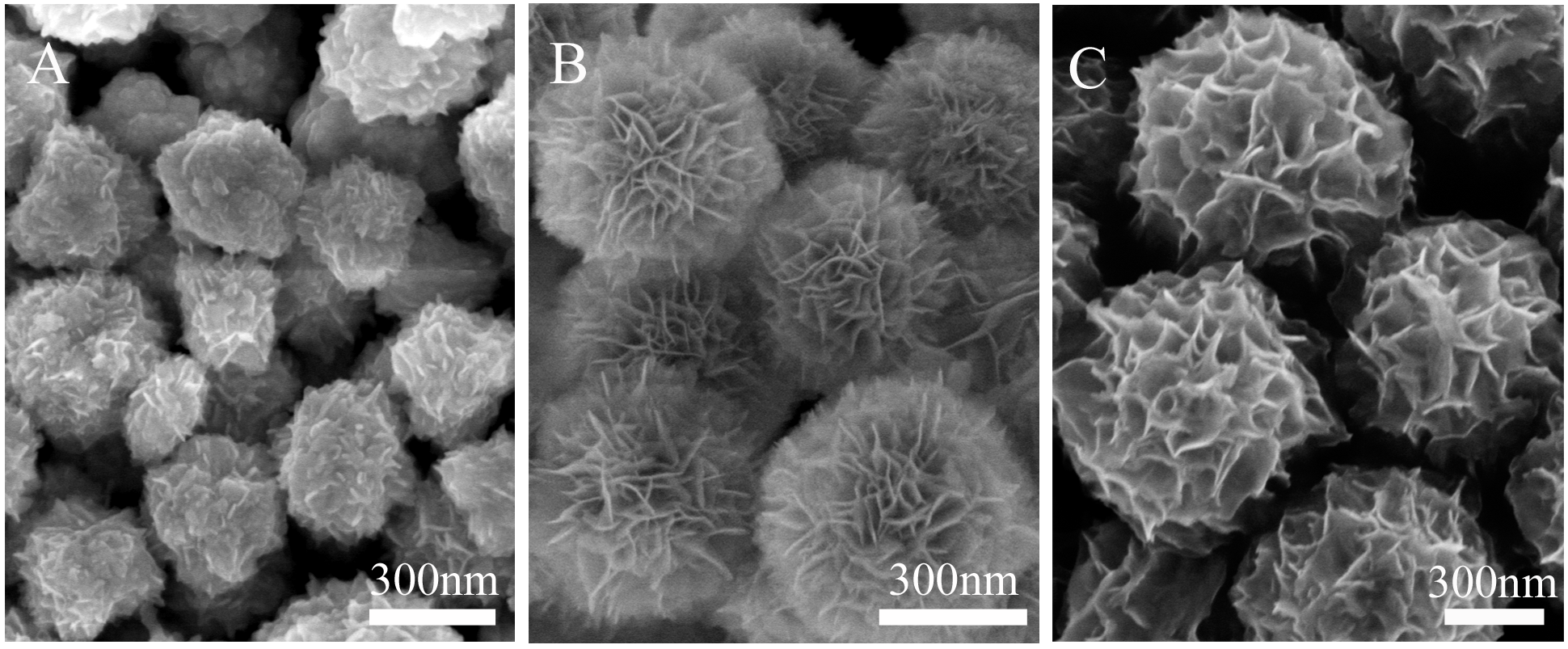


**Figure S4.** SEM images of the Bi_2_S_3_/In_2_S_3_ products with different molar ratios In-Bi-10 (A), In-Bi-30 (B) and In-Bi-50 (C).


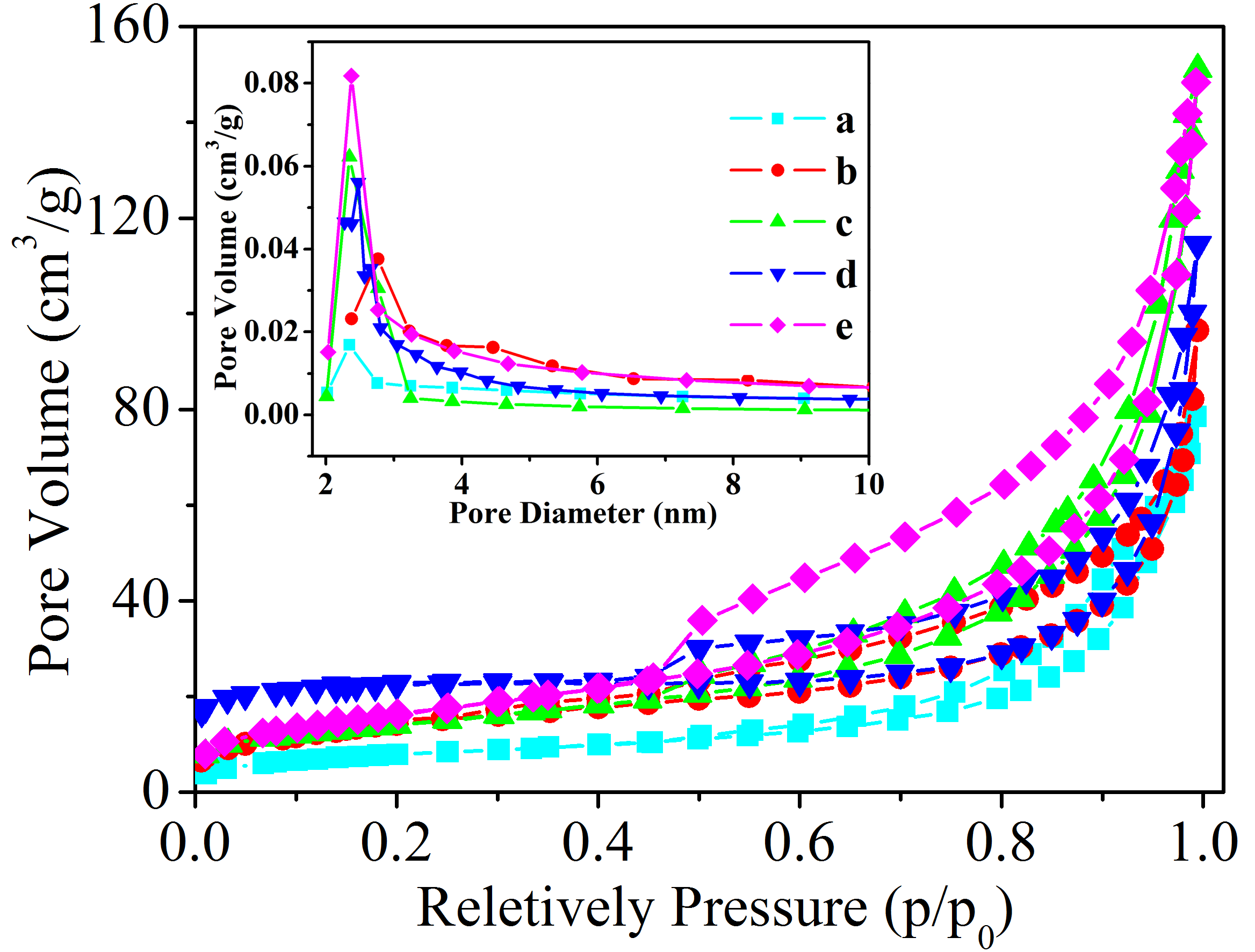


**Figure S5.** Nitrogen adsorption-desorption isotherms and the corresponding pore size distribution curves (inset) of as-prepared samples: Bi_2_S_3_ (a), In-Bi-10 (b), In-Bi-30 (c), In-Bi-50 (d) In_2_S_3_ (e).

**
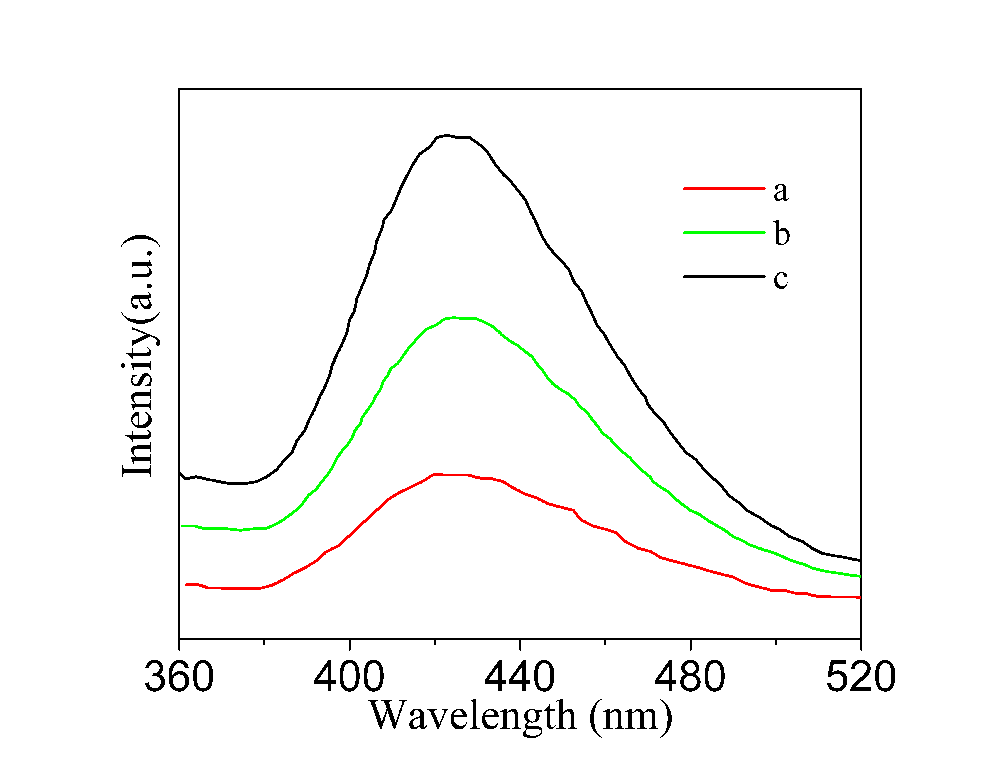
**

**Figure S6**. Fluorescence spectra of TAOH (2-hydroxyterephthalic acid) formed by the reaction of terephthalic acid (5×10^−4^ M, excitation at 315 nm) with ^●^OH radicals generated from different samples under visible-light irradiation for 15 min, Bi_2_S_3_ (a), In_2_S_3_ (b) and hierarchical Bi_2_S_3_/In_2_S_3_ (In-Bi-30) core/shell nanostructures (c).


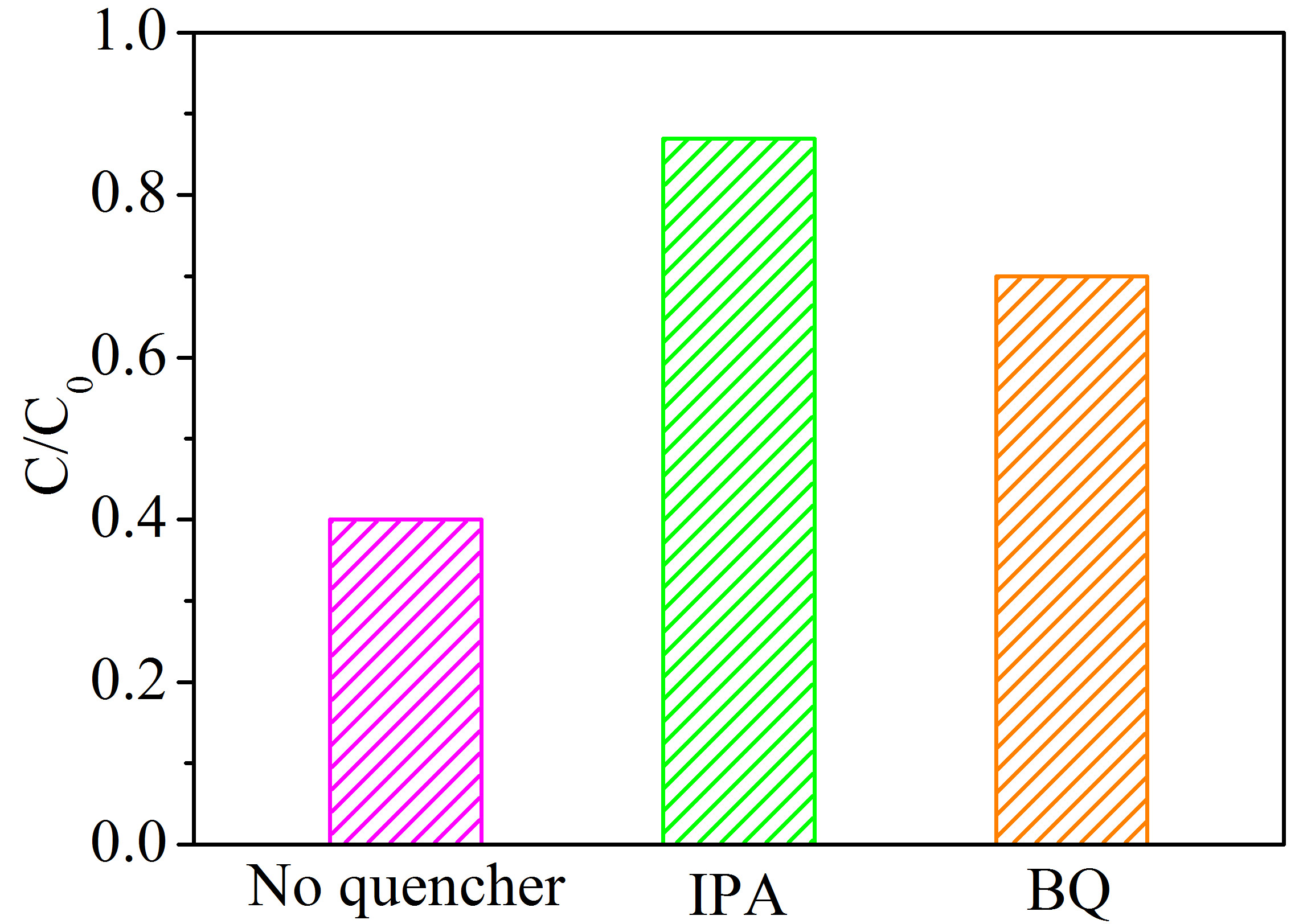


**Figure S7**. Photocatalytic activities of the hierarchical Bi_2_S_3_/In_2_S_3_ core/shell nanostructure (In-Bi-30) on the degradation of 2, 4-dichlorophenol in presence of different scavengers with 30 min visible light irradiation.


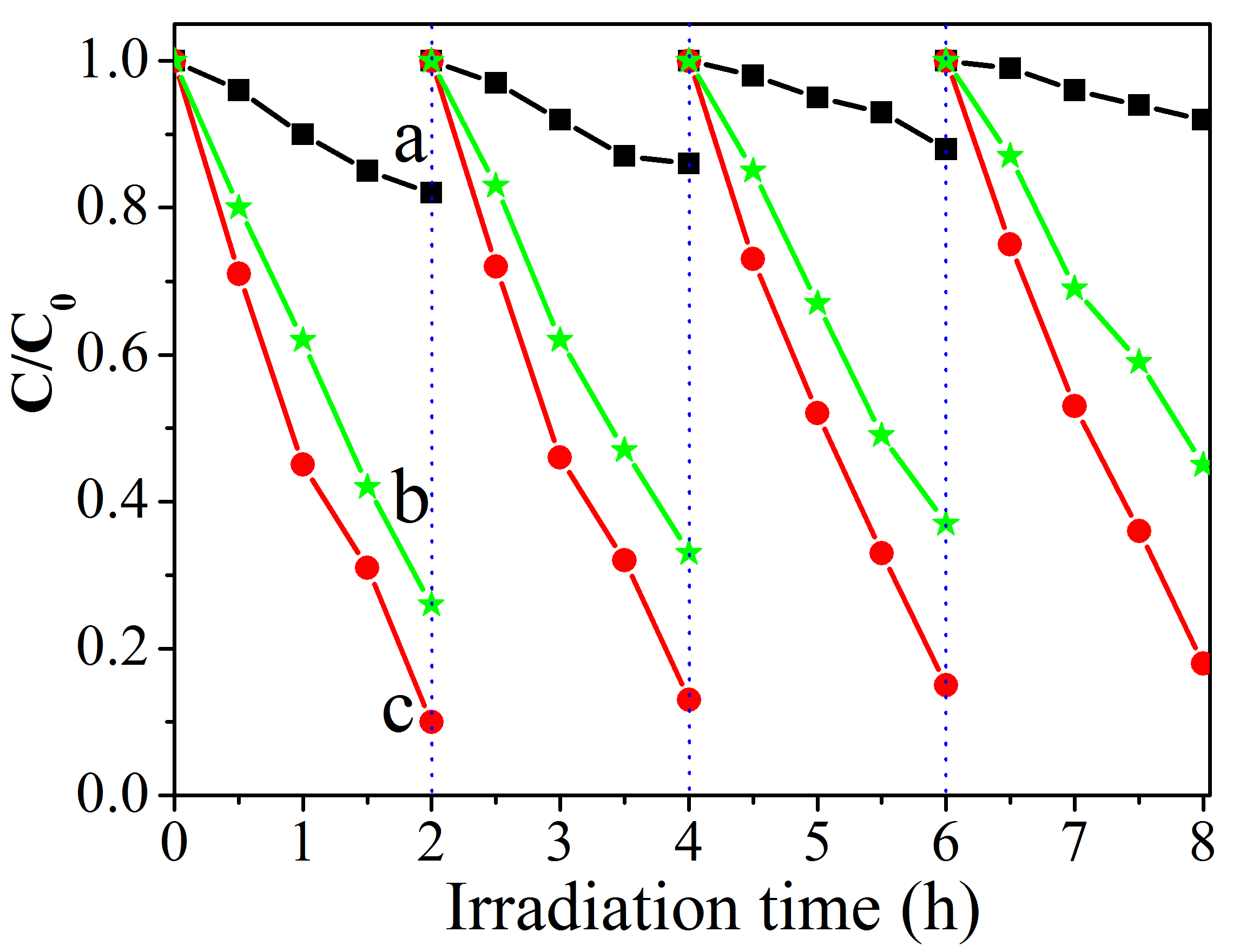


**Figure S8.** Time course of photocatalytic degradation of 2, 4-dichlorophenol over samples Bi_2_S_3_ (a), In_2_S_3_ (b) and In-Bi-30 (c).
